# Progress in the Preparation and Application of Inulin-Based Hydrogels

**DOI:** 10.3390/polym16111492

**Published:** 2024-05-24

**Authors:** Xiaoxu Liang, Danlei Lin, Wen Zhang, Shiji Chen, Hongyao Ding, Hai-Jing Zhong

**Affiliations:** 1 Foundation Department, Guangzhou Maritime University, Guangzhou 510725, China; liangxxu@126.com; 2 State Key Laboratory of Bioactive Molecules and Druggability Assessment, Jinan University, Guangzhou 510632, China; carolinelin8@163.com (D.L.); m19970042307@163.com (W.Z.); chen94599@163.com (S.C.); 3 College of Materials Science and Engineering, Nanjing Tech University, Nanjing 210009, China

**Keywords:** inulin, hydrogels, drug delivery, stimuli-responsive, biomedical applications

## Abstract

Inulin, a natural polysaccharide, has emerged as a promising precursor for the preparation of hydrogels due to its biocompatibility, biodegradability, and structural versatility. This review provides a comprehensive overview of the recent progress in the preparation, characterization, and diverse applications of inulin-based hydrogels. Different synthesis strategies, including physical methods (thermal induction and non-thermal induction), chemical methods (free-radical polymerization and chemical crosslinking), and enzymatic approaches, are discussed in detail. The unique properties of inulin-based hydrogels, such as stimuli-responsiveness, antibacterial activity, and their potential as fat replacers, are highlighted. Special emphasis is given to their promising applications in drug delivery systems, especially for colon-targeted delivery, due to the selective degradation of inulin via colonic microflora. The ability to incorporate both hydrophilic and hydrophobic drugs further expands their therapeutic potential. In addition, the applications of inulin-based hydrogels in responsive materials, the food industry, wound dressings, and tissue engineering are discussed. While significant progress has been achieved, challenges and prospects in optimizing synthesis, improving mechanical properties, and exploring new functionalities are discussed. Overall, this review highlights the remarkable properties of inulin-based hydrogels as a promising class of biomaterials with immense potential in the biomedical, pharmaceutical, and materials science fields.

## 1. Introduction

Hydrogels, three-dimensional crosslinked polymer networks with the remarkable ability to absorb and retain large amounts of water or biological fluids, have emerged as a versatile class of materials with widespread applications [1,2,3,4,5]. Their unique properties, including high hydrophilicity, biocompatibility, and soft tissue-like physical characteristics, have made them attractive candidates for diverse fields such as drug delivery systems [6,7,8], soft robotics, flexible sensors [9,10], cell scaffolds [11], wound healing [12], and tissue engineering [13].

**Figure 1:**
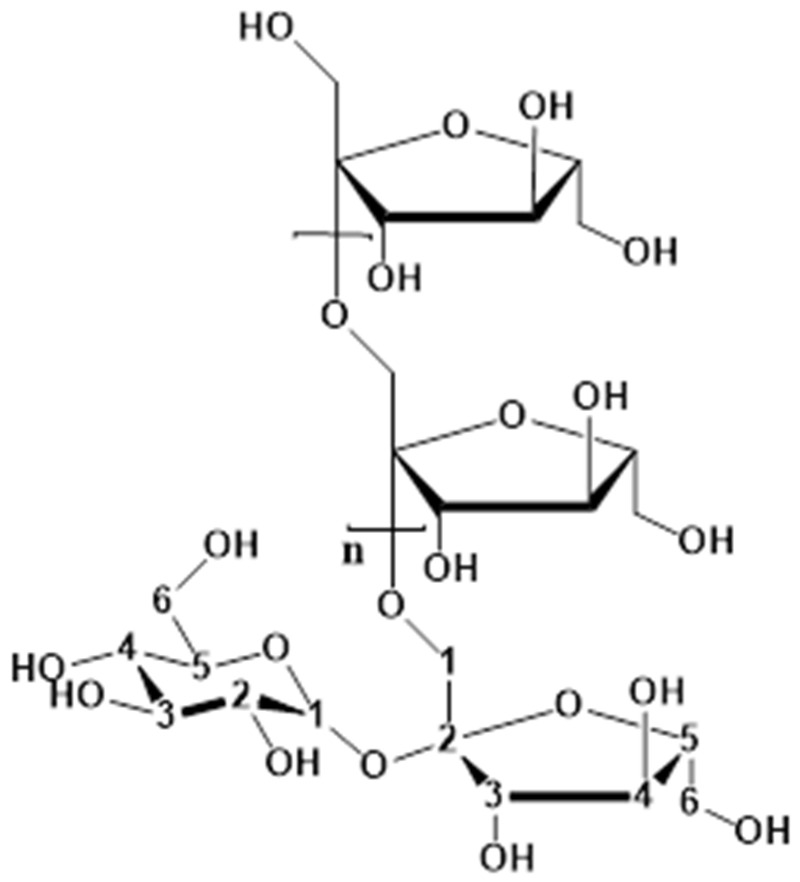
Chemical structure of inulin.

Among the various sources for hydrogel fabrication, polysaccharides have gained significant attention due to their natural abundance, biocompatibility, and structural diversity [4,14,15,16,17,18]. Inulin (its structure is shown in Figure 1), a naturally occurring polysaccharide composed of linear fructose chains with a glucose unit at the reducing end, has garnered considerable interest as a promising hydrogel precursor [19,20]. Classified as a generally recognized as safe (GRAS) reagent and a versatile dietary fiber, inulin offers several advantages, including its prebiotic properties, low caloric content, and ability to enhance food texture and mouthfeel [21,22].

The chemical structure of inulin, characterized by the absence of cyclic sugar units and the presence of *β*(2-1) glycosidic bonds, contributes to its high flexibility, chemical stability in the gastrointestinal tract (GIT), and resistance to enzymatic breakdown until it reaches the colon [23]. These unique features make inulin an excellent candidate for colon-targeted drug delivery systems and other biomedical applications. Furthermore, the rich hydroxyl groups along the inulin backbone provide sites for chemical modification, thereby enabling the tailoring of hydrogel properties and functionalities [24,25].

In recent years, the development of inulin-based hydrogels has attracted significant research interest due to their unique properties, such as biocompatibility, stimuli-responsiveness, and biodegradability [25]. Numerous reports in the literature have highlighted the potential of these hydrogels for applications in drug delivery, smart materials, and various food applications. However, a comprehensive review of the synthesis methods, properties, and applications of inulin-based hydrogels is lacking [26,27,28,29].

This review aims to bridge this gap by providing a comprehensive overview of the preparation techniques, characterization, and diverse applications of inulin-based hydrogels. The focus is on the production and utilization of inulin and its derivatives as versatile hydrogel scaffolds, with particular emphasis on their potential in drug delivery, responsive materials, and food applications. Additionally, the future prospects and challenges in the development of inulin-based hydrogels are discussed, highlighting the significant potential of this class of biomaterials for addressing various challenges in materials science and biomedical engineering.

## 2. Preparation of Inulin-Based Hydrogels

The formation of hydrogels can be achieved through physical approaches like heating, cooling, and applying shear forces; by employing chemical methods; or through a combination of both techniques to tailor the desired properties. Physical crosslinking, which is the primary mechanism in the formation of hydrogels, involves non-covalent interactions such as electrostatic forces, hydrogen bonding, hydrophobic interactions, van der Waals forces, or a combination thereof. These interactions lead to the crosslinking of polymer chains, resulting in the three-dimensional network structure characteristic of hydrogels. Alternatively, chemical crosslinking methods utilize various crosslinking agents or radiation to induce the formation of covalent bonds between polymer chains, thereby creating a chemically crosslinked network [30,31]. A summary of different methods for inulin hydrogel fabrication is shown in Table 1.

### 2.1. Physical Methods

#### 2.1.1. Thermal Induction

Thermal induction is a common and simple method for fabricating inulin gels. This method involves heating and subsequently cooling the aqueous solution, which results in a physical crosslinking network. The key mechanism behind hydrogel formation through thermal induction is the transition from a sol to a gel state. To initiate gelation, the solution containing inulin must be heated beyond the point of dissolution. When heated, the sol structure becomes disordered, causing the solution to start swelling. Cooling the solution leads to the formation of an organized polymer network [25,32]. Kim, Y et al.’s first systematic study on the mechanism of thermally induced gel inulin gel formation and proposed a possible mechanism model. The study’s results indicated that inulin gel formation occurs due to the precipitation of inulin molecules from solution into particles that form a gel, possibly through a crowding effect. The optimal conditions for gel formation are an inulin concentration of 20–30% (*w*/*v*), heating at 80–90 °C for 3–5 min at a pH of 6–8, followed by cooling to room temperature [25]. It provides a theoretical basis for inulin applications in food and other fields [25]. Han et al. produced an inulin gel by heating the inulin solution to 70 °C for 5 min and then allowing it to cool at room temperature for 5 h. The results showed that the obtained inulin gel exhibited a higher elastic modulus (*G*′) than viscous modulus (*G*″) over a wide range of frequencies, indicating a predominant elastic gel-like behavior. The rheological characterization revealed that the inulin gel had an elastic gel-like structure with shear-thinning properties suitable for oral administration. The tunable rheological properties, especially the elastic modulus, played an important role in prolonging the colonic retention of the inulin gel formulation in vivo to modulate the gut microbiome [32]. Pawel Glibowski et al. provided a more comprehensive analysis of how thermochemical treatment affects the rheological properties and structure of inulin gels. They examined the impact of various factors like pH, heating temperature, heating time, and storage temperature on the hardness and shear stress of inulin gels. The study finds that gelation depends on the presence of inulin crystallites acting as seed crystals. It elucidates how crystallite dissolution impacts gel formation. Moreover, inulin is thermally stable under neutral pH but prone to partial hydrolysis in acidic environments, affecting gel rheology and structure [33].

However, the physical crosslinked hydrogels composed solely of inulin are known to exhibit weak mechanical strength, which limits their practical application. To address this issue, it is necessary to reinforce the mechanical strength of inulin hydrogels. This effect can be achieved by adding proteins or other polysaccharides to the hydrogel, connecting these components through self-association or different interactions like hydrogen bonds, van der Waals forces, and ionic or hydrophobic interactions, ultimately enhancing the structural integrity of the hydrogels [28,34]. For example, Anna Florowska et al. modified inulin hydrogels by adding pea and soy protein, resulting in a more compact and homogeneous microstructure. As the concentration of protein increased, the hydrogels became smoother, more cohesive, and more stable, with less granularity. Notably, the hydrogels containing 3–6% plant proteins showed a significant improvement in rheological parameters such as firmness, yield stress, and spreadability [34]. In addition, Anna Florowska et al. investigated the impact of sodium alginate (SA) and chitosan (CH) on the properties of inulin hydrogels. The results indicated that the addition of SA and CH did not affect the gelation ability of the inulin solution. These modified hydrogels exhibited a more compact, smooth, and cohesive structure, as well as higher yield stress, strength, and spreadability compared to the plain inulin hydrogels. Furthermore, chitosan had a more significant impact on reinforcing the hydrogels, resulting in noteworthy increases in yield stress and strength when compared to both the inulin-only and sodium alginate-enhanced hydrogels [28]. These results suggest that adding protein polysaccharides to inulin hydrogels can create new technological properties, enhancing their application potential in various products [28,34].

#### 2.1.2. Non-Thermal Induction

Although thermal induction is a straightforward and commonly used technique for fabricating inulin hydrogels. It still has limitations, particularly when processing components with poor thermal stability. To address these issues, investigators and food manufacturers have developed alternative, non-thermal techniques which have been indicated in the previous literature and applied in inulin gelation, including ultrasonic treatment [35], high-pressure homogenization (HPH) [18], and high hydrostatic pressure (HHP) [36,37,38].

Ultrasound treatment is an environmentally friendly and sustainable method that does not require chemicals or additives, in contrast to traditional chemical/enzymatic methods [35,51,52]. Luo et al. used techniques such as FTIR, XRD, and particle size analysis to gain insights into how ultrasound affects inulin at the molecular, crystalline, and particle levels. The results indicated that ultrasound treatment alters hydrogen bonding and crystallinity without changing the molecular structure. The study found that ultrasound treatment improved the consistency, hardness, and adhesiveness of long-chain inulin gels, resulting in a finer structure. The most significant changes were observed at 1734.9 W/cm^2^ for 10 min [35].

Inulin hydrogels can also be produced using high-pressure homogenization (HPH), which applies shear forces to solutions. During the HPH process, inulin solution in a continuous flow is subjected to high pressure ranging from 50 to 400 MPa and forced through a small gap of a valve, resulting in the formation of hydrogen bonds and van der Waals interactions among dispersed particles (aggregates of molecules) [53,54]. The application of multiple physical forces increases the solubility and water retention of polymers, which benefits the gelation process and the modification of network structures [55].

Inulin gelation can also be achieved using higher pressures than those used in HPH treatment, such as high hydrostatic pressure (HHP) with an operating pressure of up to 1000 MPa [36]. During the HPP treatment, non-covalent interactions (hydrogen bonds and van der Waals forces) in inulin are broken or altered, initiating the hydrogel formation, but covalent bonds suffer minimal losses [56,57]. In addition, HHP restricts molecular movement, resulting in densely packed molecules after the break of hydrogen bonding. compared with those obtained from the conventional method The obtained hydrogels have higher strength, viscoelasticity, and water retention ability [36].

Anna Florowska et al. compared the properties of inulin hydrogels obtained using non-thermal induction methods (ultrasonic, HPH, and HHP) versus the traditional thermal induction method. It was found that all methods could produce inulin hydrogels. However, non-thermal methods allowed for faster gel formation and resulted in hydrogels with improved properties compared to those formed thermally. These properties of the resulting hydrogels included improved stability, enhanced microrheological properties such as increased elasticity and viscosity, and a tendency towards the solid phase. Non-thermally produced hydrogels also exhibit a more delicate structure, with reduced firmness and increased spreadability. Furthermore, hydrogels produced using ultrasound showed darker colors and lower values in color parameters compared to those formed using the HPH and HHP techniques. High-pressure techniques showed significant potential for modifying hydrogel properties, enabling the production of gels with varied textures—from soft and spreadable to firm—depending on the applied pressure. This adaptability indicates that high-pressure methods can be applied in tailored inulin hydrogels to meet specific requirements in various food matrices [36,38]. In summary, the non-thermal induction methods generally resulted in the faster gelation, higher elasticity, higher viscosity, and lower fluidity of the inulin hydrogels compared to thermal induction.

### 2.2. Chemical Methods

The hydrogels formed via only physical crosslinking typically display poor stability and weak mechanical strength, which subsequently limits their application [4]. In contrast, hydrogels that are synthesized through chemical reactions and crosslinked mainly via covalent bonds exhibit superior stability and mechanical properties. Additionally, the functions of hydrogels can be tailored by modifying inulin and adjusting the network structure. Inulin molecules contain numerous hydroxyl groups, providing sites for chemical modification. Therefore, different chemical approaches and strategies have been developed to create stable inulin-base inulin hydrogels with customized properties [27,28,40,42,44,47].

The preparation of such inulin-based hydrogels commonly involves inulin modification and polymerization. Methacrylate-modified inulin and free-radical polymerization are the most common methods. For instance, Liesbeth Vervoort et al. synthesized methacrylated inulin (MA-inulin) by reacting inulin with glycidyl methacrylate in N,N-dimethylformamide using 4-dimethylaminopyridine as a catalyst [39]. Subsequently, Hsin-Cheng Chiu et al. developed a series of pH-responsive inulin hydrogels by copolymerizing MA-inulin with acrylic acid (AAc) in aqueous solution using ammonium peroxydisulfate (APS) and N,N,N′,N′-tetramethylethylenediamine (TMEDA) as an initiation system [40]. The incorporation of AAc into the hydrogel was confirmed via FTIR, showing a peak for the carboxylate group of AAc. Higher MA-inulin degree of substitution and lower AAc concentration led to higher AAc incorporation efficiency. The effective network density of hydrogels significantly increased due to the covalent bonding of AAc among MA moieties of MA-inulins, which was further confirmed via TGA studies [40]. Liesbeth Vervoort et al. also investigated the gelation process of methacrylated inulin (MA-IN) solutions, monitored by measuring the elastic modulus (*G*′) and viscous modulus (*G*″). Hydrogels made from MA-IN with a low degree of substitution or feed concentration exhibit nearly frequency-independent *G*′, indicating ideal network behavior with the *G*′ plateau value reflecting the equilibrium elastic modulus related to elastic chains/crosslinks. Conversely, hydrogels with higher MA-IN substitution or feed concentrations show a slight G′ decrease at lower frequencies, suggesting network imperfections such as loose ends and entanglements. As MA-IN substitution and feed concentration increase, *G*′ at low frequencies also increases, indicative of higher crosslink density due to more intermolecular crosslinking. Higher concentrations of the initiator (APS/TMEDA) result in lower *G*′ values, likely from increased radical activity leading to reduced vinyl group conversion and fewer crosslinks. Overall, higher inulin concentrations led to faster gelation and more rigid hydrogel networks due to increased intermolecular crosslinking, but also more network imperfections like entanglements and loose ends that could relax and influence the rheological properties [41].

**Figure 2:**
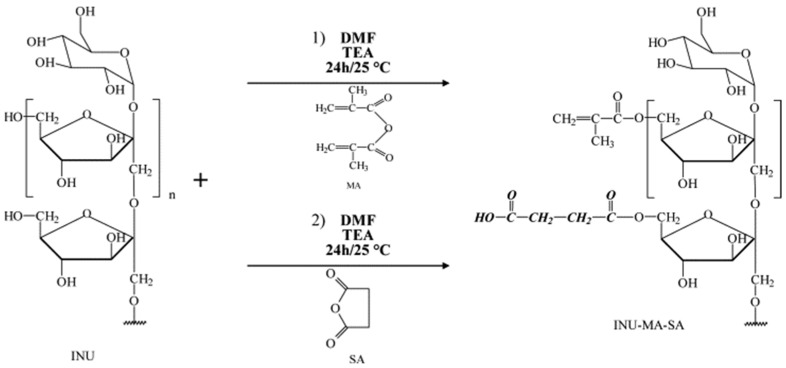
Scheme of reaction between inulin (INU), methacrylic anhydride (MA), and succinic anhydride (SA) [51].

Apart from thermally initiated polymerization, ultraviolet radiation and electron beam irradiation are common preparation methods of inulin-based hydrogels. Francesco Castelli et al. developed a methacrylated/succinilated derivative (INU-MA-SA, as shown in Figure 2) using inulin reacted with methacrylic anhydride (MA) and succinic anhydride (SA), and then using UV irradiation to obtain inulin-based hydrogels. Inulin was derivatized to introduce methacrylate and succinate groups, allowing the formation of a crosslinked hydrogel with pH-responsive swelling properties. Moreover, these hydrogels were fabricated in the absence of toxic initiators, which is beneficial for their application in drug delivery [43]. Florinella Muñoz Bisesti et al. reported on the development of two new inulin hydrogels (inulin-acrylamide and inulin-polyvinyl alcohol hydrogels, coded Inu-AAm and Inu-PVA) using electron beam irradiation. This method allows for the preparation of inulin hydrogels without the modification of inulin and in the absence of toxic initiators or chemical crosslinkers. This is a significant advancement in the field as this method has not been reported before. The thermal stability of Inu-AAm hydrogel was found to be higher than that of the precursors, while Inu-PVA hydrogel exhibited intermediate thermal stability between PVA and inulin. Furthermore, Inu-AAm hydrogel exhibited a high swelling ratio and demonstrated the ability to remove arsenic from water, suggesting the potential for further development of inulin hydrogels for environmental and other applications [44].

**Figure 3:**
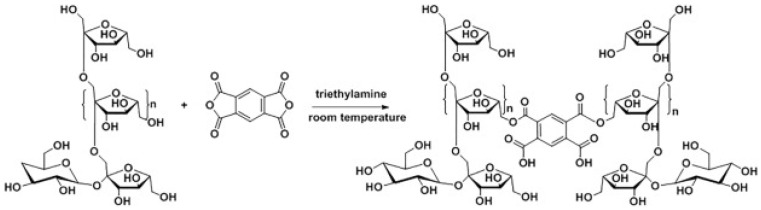
Esterification of inulin with pyromellitic dianhydride [52].

In addition to free-radical polymerization and crosslinking with acrylate functional groups, methods such as acid anhydride esterification crosslinking, Schiff base crosslinking, and click chemistry are also common methods for constructing inulin-based hydrogel crosslinking networks. For instance, Hadi Rahnama et al. developed an innovative approach by crosslinking chitosan (CS) with aldehyde-functionalized inulin (IA) [45]. This method utilizes the reactive aldehyde groups of oxidized inulin to form imine bonds with the amino groups of chitosan, enhancing the network structure of the hydrogel. The study incorporates polydopamine, which can interact with chitosan through covalent and non-covalent interactions, contributing to the stability and functionality of the hydrogel network. Moreover, the hydrogel formulation is designed to gel within 2 min under physiological conditions, which is crucial for applications requiring quick in situ settings, such as injectable drug delivery systems [45]. In addition, this innovation also addressed the limitations posed by chitosan’s low water solubility and suboptimal rheological properties, which hinder its use as an injectable hydrogel [58,59], Franklin Afinjuomo et al. developed a simplified one-pot synthesis method for creating inulin-based hydrogels crosslinked with pyromellitic dianhydride (PMDA), conducted at room temperature (as shown in Figure 3). The addition of triethylamine catalyzes the gelation process, transforming the solution into a gel in under five minutes. The esterification reaction between inulin and PMDA not only forms the hydrogel structure but also integrates carboxylic acid groups within the same step. These groups impart pH-sensitive properties to the hydrogel, crucial for applications like colon-specific drug delivery where a pH-responsive behavior is desired [46]. Giovanna Pitarresi et al. synthesize a new type of hydrogel for potential colon drug release. The study introduces a novel derivative of inulin, synthesized by derivatizing inulin with divinyl sulfone (DV) and succinic anhydride (SA). This modification of inulin is designed to enhance the reactivity of inulin towards thiol-ene conjugate addition, facilitating crosslinking with a tri-thiolated reagent (TT). The introduction of a tri-thiolated reagent for crosslinking with the INUDVSA derivative is a novel approach that contributes to the formation of a robust hydrogel structure [47]. Kazumi Izawa et al. introduced a novel glycopolymer using inulin as the scaffold, which is synthesized through a series of chemical modifications—tosylation, azidation, and Huisgen cyclocoupling—resulting in a polymer with pendent β-lactosides and terminal α-glucoside. The glycopolymer exhibits superior gelation properties compared to native inulin. It forms hydrogels at lower concentrations (critical gelation concentration, CGC), beneficial for various applications, including drug delivery systems where controlled gelling is crucial [48].

Besides the polymerizable groups, inulin can also be modified with other functional groups to expand the application of inulin hydrogels. For instance, Delia Mandracchia et al. reported the creation of an amphiphilic derivative from inulin (INU) and vitamin E (VITE), named INVITEMA, which can self-assemble into micelles and is capable of forming hydrogels through UV crosslinking. This derivative leverages the hydrophilic nature of INU and the hydrophobic properties of VITE. The structure of the nanogrid allows for a controlled and sustained release of drugs, which is crucial for chronic conditions that require consistent therapeutic levels [60].

Additionally, emulsion polymerization and core–shell structure can also be applied to the preparation of inulin-based gels. Nurettin Sahiner et al. introduced a novel method for synthesizing crosslinked inulin (X-inulin) microparticles using a water-in-oil microemulsion polymerization technique. This involves crosslinking linear inulin with divinyl sulfone (DVS) in an AOT inverse microemulsion under basic conditions. The microgels facilitate the in situ synthesis of CdS quantum dots (Q-dots), which are embedded within the microgel structure. Moreover, the microgels are used to load and release model drugs (gallic acid and caffeine), showcasing their potential as drug carriers with controlled release capabilities. This is particularly important for developing new drug delivery systems that can provide the targeted and sustained release of therapeutics [42]. Fatemeh Bahadori et al. reported a novel nano-hydrogel system that utilizes a core–shell structure, where sodium alginate forms the core and inulin forms the shell. This design is significant as it leverages the properties of both materials to enhance drug delivery. The nano-hydrogels are engineered to control the release of 5-ASA in the gastrointestinal tract. The method of synthesizing the nano-hydrogels involves a controlled gelation process using CaCl_2_, which can be tuned to adjust the gelation time and stability of the hydrogels, further optimizing the drug delivery system [49].

Enzymes are highly effective catalysts for a wide range of reactions due to their high reaction specificity under mild conditions. Additionally, enzymatic reactions are largely regioselective. This allows for the production of hydrogels with varying swelling and physical properties through enzymatic modification [50,61,62]. Christian V. Stevens et al. reported the successful enzymatic modification of inulin, a polysaccharide, using a Proleather FG-F enzyme to acylate inulin with divinyl adipate in a 100% DMF (Dimethylformamide) solution. This process resulted in the incorporation of vinyl groups into the inulin backbone and the modified inulin (Inul-VA) was obtained. Inul-VA was used to prepare hydrogels through free-radical polymerization. These gels demonstrated swelling ratios with equilibrium values of up to approximately 20, and their pore sizes ranged from 19 to 57 Å. The study established a correlation between the swelling characteristics and the mesh size of the gels, which suggests potential applications in controlled drug release systems, particularly for colon-specific drug delivery [50].

Overall, inulin-based hydrogels fabricated through chemical methods can achieve a more stable network structure and additional functionalities. Nevertheless, compared to physical crosslinked inulin-based hydrogels, the chemical modification of inulin and the chemical synthesis process of inulin-based hydrogels may potential toxicity issues or affect the biocompatibility of the resulting hydrogels. Moreover, the chemical synthesis process is also more complex than the physical methods. To address these potential disadvantages, further research and development is necessary to optimize the synthesis process.

## 3. Properties and Applications of Multifunctional Inulin-Based Hydrogels

Inulin-based hydrogels have demonstrated great versatility and potential in various practical applications. This section primarily focuses on their applications in responsive, antibacterial, and drug delivery systems.

### 3.1. Stimuli-Responsive Materials

Normally, hydrogels with special structures can be responsive to external stimulation, such as changes in temperature, pH-responsiveness, and ion strength. Among these are some hydrogels that undergo several reversible polymer network folding processes, resulting in shape changes [29,63,64,65,66]. There has been significant interest in fabricating stimuli-responsive hydrogels based on inulin, which have gained fruitful progress [29].

**Figure 4:**
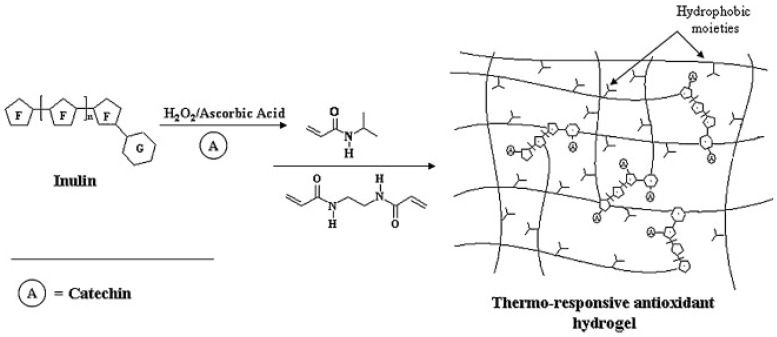
Schematic representation of the synthesis of thermo-responsive antioxidant–inulin conjugate [29].

Temperature is a common physical parameter that can cause a reversible and discontinuous volume phase change in a polymeric network. Poly(N-isopropylacrylamide) (PNIPAAm) is one important class of temperature-responsive materials composed of alkyl acrylamide polymers. PNIPAAm exhibits a sharp coil–globule transition and phase separation at its lower critical solution temperature (LCST) in water [67,68]. For instance, Spizzirri, UG et al. synthesize a series of thermo-sensitive antioxidant hydrogels by integrating catechin, an antioxidant molecule, and NIPAAm, a thermo-responsive monomer, into inulin through a free radical grafting reaction (as shown in Figure 4). Such thermo-responsive antioxidant–polysaccharide conjugates showed transition temperatures in the range of 31.3–33.1 °C and a water affinity depending on the temperature of the surrounding medium. This property is particularly useful for applications requiring temperature-sensitive activation or release mechanisms [29].

pH-responsive hydrogels are another class of “smart” materials that can respond reversibly to changes in the external pH. These hydrogels typically contain weak acid or weak base groups, which can be affected by the pH of the solution they are in. As a result, the hydrogels undergo a volume transition from a collapsed to an expanded state due to osmotic pressure. This reversible swelling–shrinking property makes these materials useful in a wide range of applications such as drug delivery systems and chemical sensors. There are also considerable reports on inulin-based smart pH-responsive hydrogels using different strategies. For instance, Giovanna Pitarresi et al. introduced novel inulin-based hydrogels (INUDVSA-TT hydrogels). Firstly, inulin was reacted with succinic anhydride (SA) to graft succinated groups on its backbone, resulting in the final hydrogel with pH sensitivity. Their ability to swell more at higher pH levels and remain stable in acidic conditions ensures that they can protect and appropriately release drug molecules in the varying pH environments of the gastrointestinal tract [47]. Hsin-Cheng Chiu et al. presented another approach to produce inulin-based smart pH-responsive hydrogels. They first prepared a methacrylated inulin derivative (MA-inulin), which was then copolymerized with acrylic acid (AAc) in an aqueous solution using free-radical copolymerization. The results indicated that the variation in hydrogel swelling in response to a pH shift from 7.4 to 2.2 became more pronounced with higher AAc content. This makes them potentially useful for applications such as colon-specific drug delivery systems [40]. Franklin Afinjuomo et al. introduced a simplified one-pot synthesis method for creating inulin-based pH-responsive hydrogels. The esterification reaction between inulin and PMDA not only forms the hydrogel structure but also integrates carboxylic acid groups within the same step. The hydrogel exhibits pH-sensitive swelling characteristics, with significantly reduced swelling at lower pH levels (from pH 7.4 to pH 1.2). This property is particularly valuable for pharmaceutical applications aiming to protect drugs from the acidic environment of the stomach and release them in the more neutral pH of the colon [46].

### 3.2. Food Industry

Food products frequently harbor agents that cause spoilage and carry pathogenic microorganisms. In recent years, microbial contamination has emerged as the leading reason for the decline in food quality, increased losses, and the spread of foodborne illnesses, raising considerable alarm. Hydrogels, which are three-dimensional network structures created by crosslinking polymers, are commonly utilized as a delivery system for active substances in food preservation, thanks to their superior ability to retain water. Developing hydrogel films that possess spectral bacteriostatic properties represents a promising strategy to improve food safety and quality [69,70].

Inulin-based hydrogel is widely used in the food and pharmaceutical industries as an excellent carrier for active substances due to its outstanding permeability, biodegradability, and reproducibility [5,71]. Wang et al. developed a hydrogel film (CMCS/inulin@Durancin GL hydrogel) with antibacterial properties and controlled release features. The film is made by grafting carboxymethyl chitosan (CMCS) onto a bacteriocin, durancin GL, and inulin using a specific chemical reaction. Increasing the concentration of durancin GL in the hydrogel increased its hydrophobicity and extended the release duration of active substances. The hydrogel film demonstrated significant antibacterial activity, particularly evident in experiments preserving salmon. Therefore, they show promise as fresh-keeping packaging materials for practical use [69].

There is a growing concern about the health implications of high-fat foods, such as obesity and chronic diseases, leading to an increased interest in developing healthier dietary options. Sausages are a popular food worldwide due to their favorable nutrients, shelf-life stability, and convenience. However, they typically contain high levels of dietary fat, which contributes to their characteristic flavors and smooth mouthfeel. To address the negative health impacts associated with high-fat content, research has been conducted into fat replacement strategies [72,73].

Inulin, known for its low-calorie content, mild sweetness, and robust tolerance to gastric acid, presents a promising substitute for sugars and fats, offering significant nutritional advantages [74,75]. Li et al. introduced a hybrid hydrogel made from inulin and microcrystalline cellulose (IMC gel) to reduced-fat pork sausage formulations, significantly improving several properties of the sausage. Specifically, IMC gel enhances textural properties such as hardness and chewiness, increases water-holding capacity, and improves oxidative stability by increasing the metmyoglobin content and thiobarbituric acid reactive substance (TBARS) value while decreasing the peroxide value (POV). Additionally, IMC gel influences the molecular structure by decreasing α-helix conformation and increasing β-fold conformation, which contributes to the formation of a dense and porous gel network structure. The research suggests that IMC gel can be a beneficial fat replacer in sausage formulations, potentially leading to healthier reduced-fat sausage products without compromising quality [73].

### 3.3. Wound Dressing and Tissue Engineering

Wound management remains a critical challenge in human healthcare and necessitates a multidisciplinary approach [76,77,78]. Developing hybrid biomaterials that possess antioxidant, anti-inflammatory, antibacterial, and other crucial properties is vital for promoting rapid and effective wound healing [79]. Inulin stands out due to its biodegradability, biocompatibility, non-toxicity, and hydrophilicity, making it a promising candidate for use in wound dressings [80].

**Figure 5:**
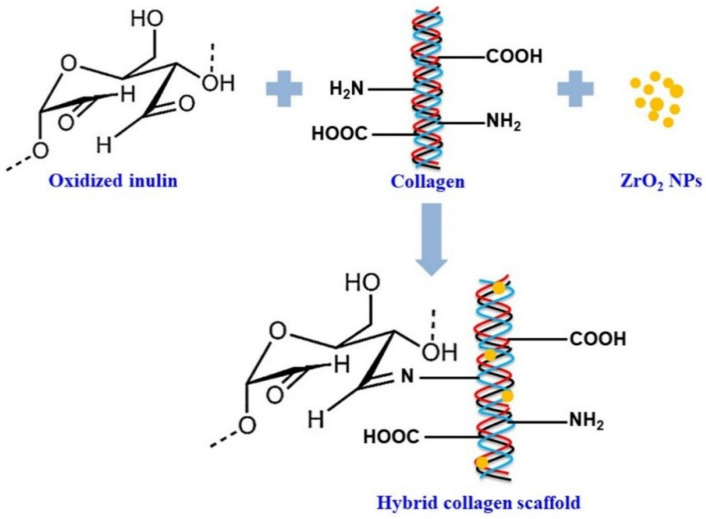
Schematic presentation of the interactions between collagen and oxidized inulin and ZrO_2_ nanoparticles [81].

Cheirmadurai Kalirajan et al. reported the creation of hybrid collagen scaffolds enhanced with oxidized inulin and ZrO_2_ nanoparticles for biomedical use (as shown in Figure 5). These scaffolds exhibit essential biomaterial characteristics such as biodegradability, porosity, swelling ability, and both enzymatic and thermal stability, with hydrothermal stability being improved up to 96 °C. They are biocompatible with stem and osteoblast cells. The scratch wound healing assay showed that these scaffolds can heal wounds by up to 60% after 24 h of incubation, displaying a higher cell migration index than native collagen scaffolds. These findings indicate that the hybrid collagen scaffolds are suitable for wound dressing and tissue engineering applications [81].

### 3.4. Drug Delivery System

The remarkable swelling capacity, permeability, and biodegradability of inulin-based hydrogels position them as ideal candidates for drug delivery systems [82,83]. Furthermore, these inulin hydrogels can undergo chemical modifications to boost their drug delivery effectiveness, making them suitable scaffolds for various drug delivery applications [84]. In comparison to other polysaccharide-based hydrogels, inulin-based hydrogels typically exhibit shear-thinning properties, which renders them advantageous for further application as injectable and oral administration drug delivery systems [58,59,85,86,87,88]. Additionally, inulin’s capability for enzymatic degradation specifically in the colon enhances its utility for colon-targeted drug delivery. It can transit through the upper gastrointestinal tract intact and reach the colon, where it is broken down by specific bacteria, thereby releasing the drug at the targeted site [82]. Some representative inulin-based hydrogel drug delivery systems are summarized in Table 2.

There are primarily two methods to load drugs into inulin-based hydrogels: (1) encapsulating the drug during the gel crosslinking process and (2) allowing the drug to diffuse into the spaces of the swollen hydrogel after the crosslinking process. However, when using the encapsulation method, it is challenging to remove unreacted chemicals from the hydrogel; the diffusion method requires placing the hydrogel in a drug solution and allowing it to swell to equilibrium, which is time-consuming and inefficient [21,92]. For instance, Guy Van den Mooter et al. explored the two distinct methods for loading proteins into methacrylated inulin (MA-IN) hydrogels. The results indicated that proteins are more uniformly distributed within the hydrogel when loaded during formation, leading to more consistent release kinetics. In contrast, post-formation loading often results in protein accumulation near the hydrogel surfaces, leading to quicker release. Additionally, loading during hydrogel formation provides better control over the release rates and profiles compared to loading after formation. It allows for a more sustained release, avoiding the rapid burst release seen with post-formation loading. The choice of method may depend on the specific application and required release profile [21,89]. Norsyazwani Solehah Norudin et al. investigated the relationship between encapsulation efficiency and inulin concentration in inulin-based hydrogels. The study found that as the concentration of inulin increased from 5% to 15% (*w*/*v*), there was a corresponding increase in the protein encapsulation efficiency. This can be attributed to the stronger interaction between alginate and inulin, which then leads to a higher encapsulation yield. The findings suggest that the presence of inulin not only enhances the stability of the hydrogel matrix but also improves its capacity to encapsulate and retain a greater amount of protein [91].

**Figure 6:**
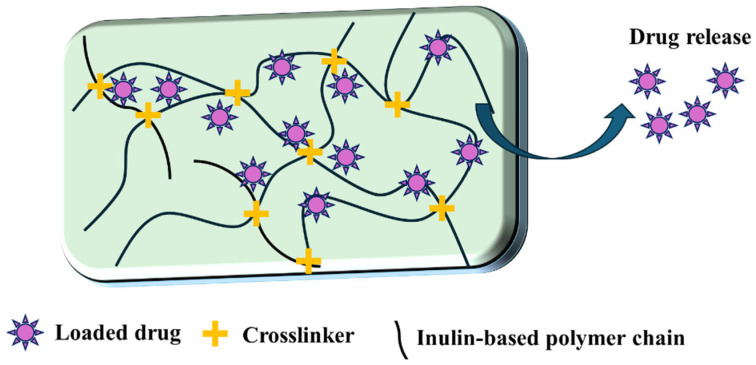
Release mechanism of loaded drugs using inulin-based hydrogel.

The release of drugs from hydrogels is a complex process, with various release mechanisms proposed by researchers (Figure 6). The three widely recognized release mechanisms are diffusion, swelling-controlled release, and chemically controlled release. These mechanisms protect the drug from degradation in the upper gastrointestinal tract and ensure controlled release at the targeted site, such as the colon. The release mechanism of the drug can be affected by various factors, including the composition of the hydrogel, the molecular weight of the drug, the degree of crosslinking, and the environmental conditions [27,89,93]. Norsyazwani Solehah Norudin et al. also investigated the mechanism of the controlled pH-sensitive release of inulin-based hydrogels. The hydrogels’ network structure, determined via the crosslinking and interaction between alginate and inulin, as well as the presence of calcium ions acting as a crosslinker, plays a crucial role in controlling the release behavior of the encapsulated proteins in different pH environments. The encapsulated protein is protected in the acidic environment of the stomach (pH 1.2) by minimizing its release. It is then rapidly and completely released (within 90 min) in the alkaline environment of the small intestine (pH 7.4). This ability to adapt to the pH changes from the stomach to the intestine ensures that the protein is protected during its passage through the stomach and is efficiently released when it reaches the more favorable conditions of the small intestine. The targeted release mechanism is essential for maximizing the therapeutic effectiveness of the encapsulated protein drugs [91].

After modification, the inulin derivative can adjust the polymer network structure of the hydrogel and control the release behavior of the drug. Giovanna et al. synthesized a novel inulin-based hydrogel by crosslinking an inulin divinylsulfone derivative (INUDV) and O,O′-bis(2-aminoethyl)poly(ethylene glycol) (PEGBa). This hydrogel enables the sustained release of flutamide, potentially decreasing the need for frequent daily dosages. Such sustained release could enhance therapeutic effectiveness and improve patient adherence. Additionally, the hydrogel possesses mucoadhesive properties, allowing it to stick to mucosal surfaces within the gastrointestinal tract. This characteristic helps extend the drug’s retention time in the body, improving its absorption and overall bioavailability. Consequently, the hydrogel formulation extends the half-life of flutamide and enhances its bioavailability compared to traditional tablet forms. This enhancement could lead to more efficient drug delivery and better therapeutic results [90]. Fatemeh Bahadori et al. realized the protection and transportation of drugs by constructing a core–shell nano-hydrogel oral nano-drug delivery system (NDDS), where 5-ASA-loaded sodium alginate (Na-Alg) forms the core and quaternized inulin (QIn) forms the shell. The crosslinked Na-Alg core serves to protect 5-ASA from the acidic pH of the stomach, while the quaternized inulin shell, known for its strong mucoadhesive properties, facilitates digestion by the intestinal flora, thereby providing the controlled release of 5-ASA at the site of inflammation in the small and/or large intestine. This specificity is advantageous for treating colon-specific diseases such as inflammatory bowel disease (IBD) [49]. Delia Mandracchia et al. obtained INUPAHy hydrogels via crosslinked succinic derivatives of inulin (INU-SA) with α,β-polyaspartylhydrazide (PAHy). The hydrogels were designed to release these therapeutic agents in a controlled manner within the colon. The drug release profiles showed that the hydrogels exhibited pH-sensitive swelling behavior, releasing a higher number of drugs in simulated intestinal fluid compared to gastric fluid. This controlled release pattern suggests that these hydrogels have the potential for the oral administration of glutathione (GSH) and oxytocin (OT), providing a promising approach for treating inflammatory bowel disease [27].

The drug release behavior can be tuned by varying the crosslinker ratio [49,80]. Afinjuomo et al. synthesized inulin-based hydrogels crosslinking oxidized inulin with adipic acid dihydrazide (AAD). The drug delivery ability of the resulting hydrogels was investigated using 5FU as the model drug for in vitro release. The release profile was influenced by both the pH of the release medium and the crosslinking density. The hydrogels showed higher release rates in acidic pH, with about 85% of 5FU released within 2 days at pH 5.0. The study showed that the release of 5FU was dependent on the crosslinking density. Higher concentrations of the crosslinker resulted in slower release rates. These inulin hydrogels have potential as drug delivery systems for colon-targeted drug delivery, especially for treating colon cancer [80].

Hydrogels and polymeric micelles are commonly used materials in drug delivery systems [6,17,60,94,95,96]. Hydrogels are suitable for loading hydrophilic drugs, while polymeric micelles are better for encapsulating hydrophobic drugs. However, in many medical conditions, especially chronic diseases like Crohn’s disease, a combination of drugs with different solubility profiles is often required for effective treatment. Dual delivery systems allow for the simultaneous administration of both hydrophilic and hydrophobic drugs, enhancing the therapeutic outcomes [7]. For instance, Delia Mandracchia et al. created an amphiphilic derivative from inulin (INU) and vitamin E (VITE), named INVITEMA, which can self-assemble into micelles and is capable of forming hydrogels through UV crosslinking. This derivative utilizes the hydrophilic nature of INU and the hydrophobic properties of VITE to embed the micelles within a hydrophilic polymer matrix. This creates a network that can effectively load and release both hydrophilic and hydrophobic drugs. After 120 h, approximately 60% of the loaded beclomethasone dipropionate (BDP) was released from the INVITEMA3/2 h nanogrids, demonstrating a sustained release profile over the testing period. The study results demonstrate the system’s capability to deliver both hydrophilic and hydrophobic drugs in a targeted and efficient manner, especially for colon drug delivery applications [60].

Inulin hydrogel can serve as a drug carrier and also enhance bio-adhesion, prolong color retention, and regulate gut microbiota [32]. Inflammatory bowel disease (IBD) is a chronic and refractory condition characterized by a disrupted epithelial barrier, dysregulated immune balance, and altered gut microbiota [97,98]. Nano-enabled interventions have the potential to restore gut homeostasis and alleviate inflammation in IBD. For instance, Zhang et al. developed a novel composite for treating IBD. The hydrogels combine olsalazine-based nanoneedles with microbiota-regulating inulin hydrogel Cu_2_(Olsa)/Gel. The composite exhibits ROS-scavenging abilities and anti-inflammatory effects, which are essential for alleviating inflammation in IBD. Additionally, the composite’s macroporous structure, improved bio-adhesion, and enhanced colon retention after oral administration provide targeted drug delivery to the affected area. The study suggests that a composite of a self-delivering nanodrug and dietary fiber hydrogel can be effective at reshaping intestinal homeostasis and offering a potential treatment for IBD [87].

A key requirement for the application of hydrogels in medical treatments is their biodegradability. For a hydrogel used as a drug delivery system, it is important that it be safely and completely cleaned from the human body post-delivery. Inulin is particularly suitable for targeting the colon due to the specific degradation of its glycosidic bonds by the colonic microflora. Importantly, the degradation behavior of modified inulin derivatives, which are used to form hydrogels, should be thoroughly examined by exposing them to inulinase or cecal contents to ensure their safe decomposition [43,90]. For instance, Francesco Castelli et al. investigated the degradation of inulin-based hydrogel (INU-MA-SA-based hydrogels). The enzymatic degradation studies revealed that both the inulin-based derivative and the hydrogel underwent degradation via inulinase. The hydrogel showed a degradation rate of 53 ± 3% (*w*/*w*), indicating its susceptibility to enzymatic breakdown. Giovanna Pitarresi et al. detected the total amount of fructose in each sample to evaluate the degradation of inulin-based hydrogels. The results demonstrate that both INUDVSA-TT 8 h and INUDVSA-TT 24 h hydrogels undergo a pronounced degradation due to the enzyme [47,90]. Overall, these findings highlight the potential of inulin-based hydrogels as a biodegradable drug delivery system in the pharmaceutical sciences field.

In summary, inulin-based hydrogels facilitate controlled, targeted, and sustained drug release, making them promising candidates for the development of advanced and effective drug delivery systems, especially for oral, colon-targeted, and inflammatory conditions like IBD.

## 4. Conclusions

In recent years, inulin-based hydrogels have demonstrated remarkable versatility in diverse applications due to their unique properties derived from the natural polysaccharide inulin. These include biocompatibility, biodegradability, and the ability to tailor functionalities through chemical modifications. Various synthesis methods, both physical and chemical, have been developed to prepare inulin-based hydrogels with desired properties. Their stimuli-responsive behavior, antibacterial activity, and potential as fat replacers make them attractive for applications in drug delivery, smart materials, and the food industry. Notably, inulin-based hydrogels show significant potential for colon-targeted drug delivery due to their selective degradation in the colon, making them ideal carriers for the treatment of colon-specific diseases. Their ability to load both hydrophilic and hydrophobic drugs further expands their therapeutic potential. While significant advancements have been made, several challenges remain to be addressed. Optimizing synthesis processes to enhance mechanical properties and fine-tune stimuli-responsiveness is crucial for practical applications. Comprehensive studies on the biodegradability and safety of chemically modified inulin derivatives are essential for their successful clinical translation. Moreover, the development of inulin-based hydrogels with advanced functionalities, such as incorporating antimicrobial agents, cell-instructive cues, or multi-responsive behavior, could further expand their applications in tissue engineering, wound healing, regenerative medicine, wearable sensors, and artificial skin.

In conclusion, inulin-based hydrogels represent a promising and versatile class of biomaterials with immense potential for various applications, particularly in the biomedical and pharmaceutical fields. With continued research and development, these hydrogels could lead to innovative solutions for drug delivery, tissue engineering, and other healthcare challenges, ultimately improving patient outcomes and quality of life.

## Figures and Tables

**Table 1:** Summary of different methods for inulin hydrogel fabrication.

Method		Advantages	Disadvantages	Ref.
Physical Methods	Thermal Induction	Simple, straightforward	Weak mechanical strength	[25,28,32,33,34]
	Non-Thermal Induction (Ultrasound, HPH, HHP)	Avoids thermal degradation, higher elasticity, higher viscosity, and lower fluidity	Requires specialized equipment; the operation may be more complex	[18,35,36,37,38]
Chemical Methods	Free-Radical Polymerization (with methacrylated inulin)	Stable hydrogels with improved mechanical properties via covalent crosslinking	Involves chemical modification of inulin; may raise biocompatibility concerns	[39,40,41,42]
	Radiation Crosslinking (UV, electron beam)	Avoids the use of toxic initiators; simple process; and stable hydrogels with improved mechanical properties via covalent crosslinking	Requires specialized equipment; potential radiation damage	[43,44]
	Other Chemical Crosslinking (click chemistry, Schiff base, esterification)	Allows introduction of various functional groups, tunable properties like pH sensitivity	Complex synthesis; potential toxicity issues from reagents/initiators	[42,45,46,47,48,49]
	Enzymatic synthesis of inulin derivative	Highly specific, regioselective, mild reaction conditions	Limited availability and high cost of enzymes	[50]

**Table 2:** Summary of representative inulin-based hydrogel drug delivery system.

Drug Delivery System	Model Drug or Protein	Advantages and Contributions	Disadvantages and Limitations	Ref. No
Cu2(Olsa) Gel	- Olsa - Cu2(Olsa) nanoneedles embedded in inulin hydrogel.	Cu_2_(Olsa) gel enhances inflammation alleviation and immune modulation. Improved tissue repair, mucus secretion, and tight junction protein expression.	The synthesis of the MOF nanoneedle requires a hydrothermal method, which may limit large-scale production compared to some other nanoparticle synthesis methods.	[87]
MA-IN Hydrogel	Bovine serum albumin (BSA)	Methacrylated hydrogels offer controlled protein release for colon-specific drug delivery. Protein release is influenced by factors like drug loading method and molecular weight. Inulin hydrogels show biodegradable properties suitable for colon-specific drug delivery.	The optimal pH used for studying inulinase degradation (pH 4.7) is not typical of the colon environment, which could affect the relevance of those specific results.	[89]
INUDV/PEGBa hydrogel	Flutamide	Extended drug release, improved bioavailability, and enhanced patient compliance. Resistance to hydrolysis in gastric conditions and mucoadhesive properties. Oral dosage form for flutamide with reduced side effects.	The presence of the hydrophobic drug flutamide reduces the swelling ability of the hydrogel in aqueous media compared to the unloaded hydrogel.	[90]
INUPAHy hydrogels.	Glutathione (GSH) Oxytocin (OT)	Potential use for oral treatment of inflammatory bowel disease. Water-soluble derivatives with pH-dependent swelling properties. Degradable hydrogels suitable for colonic drug delivery. Controlled release of model drugs glutathione and oxytocin, with higher release at intestinal pH compared to gastric pH.	Complex chemical synthesis. no discussion includes scale-up challenges, long-term stability, and in vivo performance.	[27]
INVITEMA micellar-hydrogel nanogrids	beclomethasone dipropionate (BDP)	Allows co-delivery of hydrophilic and hydrophobic drugs in a single system for combination therapy. Utilizes biocompatible and biodegradable materials like inulin and vitamin E. Inulin is specifically degraded in the colon, making it suitable for colon-targeted drug delivery. UV crosslinking method avoids the need for radical initiators and provides sterilization. Demonstrated controlled release of the hydrophobic model drug beclomethasone dipropionate	Loading of hydrophilic drugs like curcumin before UV crosslinking led to degradation, so alternative loading methods may be required. Complexity of the multi-component system and synthetic process.	[60]
INUAAD Hydrogels	5-fluorouracil (5FU)	Fabricated by crosslinking oxidized inulin with adipic acid dihydrazide (AAD) without using any catalyst or organic solvent. Rapid (2–4 min) formation under physiological conditions (pH 7.4). pH-sensitive drug release behavior, releasing 5FU faster at acidic pH compared to physiological pH. Good biocompatibility with negligible cytotoxicity against HCT116 colon cancer cells.	The burst release of 5FU from the hydrogels was quite high initially, which may need to be controlled for sustained drug delivery applications.	[80]
CS/DA/IA hydrogel	Indomethacin, Dopamine	Simple and green preparation method without using toxic catalysts or initiators. Injectable and rapid gelation (within 2 min) suitable for minimally invasive applications. Tunable properties like porosity, swelling, and degradation rate by varying inulin aldehyde content. pH-responsive sustained drug release behavior, allowing localized delivery. Good biocompatibility and no cytotoxicity against L-929 fibroblast cells.	Chemical modifications and polymer additions are needed to enhance chitosan properties.	[45]
Sodium alginate-inulin hydrogel	Bovine serum albumin (BSA)	Alginate-inulin hydrogels protect protein drugs in the stomach and release them in the intestine. Improved protein encapsulation efficiency with increased inulin amount in hydrogels. pH-sensitive swelling behavior of hydrogels, minor swelling in acidic pH. Alginate and inulin combination controls protein release in the gastrointestinal tract.	High concentrations of alginate and inulin (beyond those tested) did not produce well-formed hydrogel beads, limiting the maximum loadings that could be achieved.	[91]

## Data Availability

Not applicable.
